# Rapid Griess assay (RGA): a chairside test for *ex vivo* semi-quantitative oral nitrite measurement and *in vitro* assessment of nitrite production by oral bacteria

**DOI:** 10.1080/20002297.2025.2517039

**Published:** 2025-06-12

**Authors:** Simeon K B Mavropoulos, Rabi Zaiton, Amina Basic, Gunnar Dahlén

**Affiliations:** a Institute of Odontology, Sahlgrenska Academy, Department of Oral Microbiology and Immunology, University of Gothenburg, Gothenburg, Sweden; b Swedish Public Dental Service, Region Västra Götaland, Gothenburg, Sweden

**Keywords:** Chairside test, Griess assay, nitrate, nitrite, nitric oxide, oral biomarkers, oral microbiota

## Abstract

**Background:**

Nitrite (NO_2_^−^) is produced through enzymatic reduction of dietary nitrate (NO_3_^−^) by oral bacteria: a process contributing to cardiovascular – and possibly oral – health. NO_2_^−^ quantitation in biological samples is a complex exercise, and available methods are not well-adapted for chairside use. Therefore, we aimed to develop and evaluate a semi-quantitative chairside test for NO_2_^−^ in oral samples. We also evaluated NO_2_^−^ generation in several bacterial species *in vitro*.

**Materials and methods:**

From 12 healthy individuals, tongue, saliva and plaque samples were collected and evaluated chairside across 4 weeks, using the rapid Griess assay (RGA). The RGA was further used to test bacterial species for NO_2_^−^ production.

**Results:**

In saliva, plaque and tongue samples, low, variable and high NO_2_^−^ levels, respectively, were found. Tongue samples were the most stable over time. High and medium NO_2_^−^ production capacities were shown by *Actinomyces* spp. (including *Schaalia odontolytica*), *Veillonella parvula,* and *Rothia* spp. RGA results were reproducible.

**Conclusion:**

The RGA provided stable and reliable results for chairside NO_2_^−^ semi-quantitation, and revealed elevated and stable NO_2_^−^ levels on the tongue. *In vitro*, bacterial NO_2_^−^ production was consistent with the available literature, but uncertainty remains regarding *Neisseria* spp. Our results showed promise for clinical and research applications of the RGA.

## Key messages

- The rapid Griess assay (RGA) showed promise as a chairside research tool for the exploration of oral NO_2_^−^ production.
- Oral NO_2_^−^ concentrations were highest on the tongue dorsum, and healthy individuals differed extensively in their oral NO_2_^−^ production abilities.
- The oral bacterial species *Actinomyces* spp. (including *Schaalia odontolytica*), *Rothia* spp. *Veillonella parvula* and *Aggregatibacter actinomycetemcomitans* showed NO_2_^−^ production with RGA method.

## Introduction

Interest in nitric oxide (NO) and the pathways for its production in humans have bloomed in the recent decades due to the important contribution of NO in cardiovascular health and prevention of disease by, among others, its vasodilating, metabolic, muscle relaxing and anti-diabetic properties [1,2]. Moreover, it has been suggested that this molecule or its precursors – nitrite (NO_2_^−^) and nitrate (NO_3_^−^) – may also be involved in maintaining oral health [3,4]. *In vivo*, a negative correlation between caries incidence and salivary NO_2_^−^ and NO levels has been reported [5]. Moreover, in the presence of acidified NO_2_^−^, growth potential of traditional caries-associated bacteria is reported to be markedly reduced *In vitro* [6]. Similarly, consumption of NO_3_^−^ has been shown to reduce gingival inflammation *in vivo*, and reduction of viability and counts of periodontitis-associated bacteria has been shown *in vitro*, when exposed to acidified NO_2_^−^ [7–9].

The production of NO and NO_2_^−^ in humans ensues through the well-established L-arginine pathway and the nitrate-nitrite-NO-pathway [10]. The former being an endogenous human production pathway, whereas the latter is dependent on oral bacteria [11,12].

Traditionally, quantitation of NO_2_^−^ has been performed in various liquids – commonly water, saliva, plasma and urine – using variations of the Griess diazotization reaction [13]. Different sample pre-treatments prior to analysis are usually required. Often, spectrophotometric analysis is preformed, but several other methods are also in use – e.g. gas- and liquid- chromatography, chemiluminescence and mass spectrometry [13,14].

These methods all have in common that they require expensive equipment, pre-treatment of samples, and that they are time-consuming, rendering them ill-suited for chairside application.

Results from previous studies show promise for derivatives of NO_3_^−^ as either biomarkers for oral diseases or potential therapeutic pathways addressing various diseases [1,5,7,15]. However, due to the complexity of NO_2_^−^ quantitation and lack of simple and versatile chairside methods, clinical studies and routine use become expensive, time consuming and cumbersome. Therefore, we aimed to present a simple chairside method – the rapid Griess assay (RGA) – for semi-quantitation of NO_2_^−^ in samples from saliva, dental plaque and tongue scrapings, based on the Griess diazotization reaction [16]. Further, we aimed to verify the NO_2_^−^-generating capacity in a number of oral and non-oral bacterial species.

## Materials and methods

### The rapid Griess assay (RGA)

**Figure 1.**
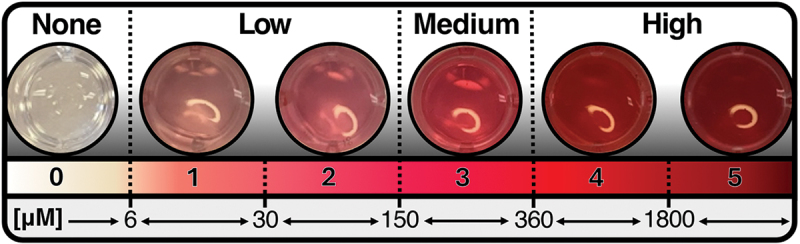
The RGA grading scale. From top to bottom: interpretation of grading, RGA grades with photos and a continuous color gradient, and the corresponding NO_2_^−^ concentration intervals.

A novel modification of the well-established Griess method for sample NO_2_^−^ semi-quantitation (RGA) was used in this study [16]. A solution of 50 μL each Sulfanilic acid and *1-Naphthylamine-7-Sulfonic acid* (reagents I and II, respectively) were added to 100 μL NO_3_^−^ broth containing 0.1% w/v KNO_3_ (Nitritreagens and Nitratbuljong, Laboratoriemedicin Sahlgrenska Universitetssjukhuset, Gothenburg, Sweden), within which participant samples or cultured samples had been incubated at room temperature for 5 min. The degree of pink and red color saturation was used for determination of sample NO_2_^−^ concentration (Figure 1). This color saturation/grading scale was based on known concentrations of NO_2_^−^ measured visually and spectrophotometrically.

### Study population and sampling

Ethical approval for this pilot study was granted by the Swedish Ethical Review Authority (Etikprövningsmyndigheten) with case number/dnr 2021–00976.

In this study, 12 volunteering healthy dental students aged 23–34 years participated (7 female, 5 male), after being verbally recruited. No screening of oral or general health was performed in the recruiting process. All participants were instructed to refrain from oral hygiene measures and consumption of foods the evening prior to as well as the day of sampling. Sample collecting was performed in the morning.

From each subject, samples were collected at four different occasions with intervals of 1 week. Three samples were taken at each sampling occasion: one from the tongue dorsum; one stimulated whole saliva sample; and one supragingival plaque sample. All samples were collected in duplicates and transported in VMGA III (Viability Medium, Göteborg, Anaerobically prepared and stored) as described by Dahlén and coworkers, and further analyzed in the microbiological laboratory [17]. Tongue samples were collected with a sterile cotton pellet held with a sterile tweezer that was rubbed for 15 s on the dorsum of the tongue. For saliva samples, subjects chewed a paraffin tablet while collecting saliva in a cup for 5 min. Thereafter, a sterile cotton pellet held with sterile tweezers was swirled in the saliva until fully saturated, and then pressed against the side of the cup to reduce the excess of saliva. Plaque samples were collected with sterile Gracey curettes at several loci throughout the mouth, until 5 μL plaque had been collected.

The samples were directly placed in VMGA III for transportation to further *in vitro* lab analysis or in NO_3_^−^ broth with reagents I and II, and then visually graded for NO_2_^−^ concentration *ex vivo*.

### Ex vivo NO_2_^−^ production

Recording of the RGA grade was performed within 10 min after sample application to the RGA-reagents by two clinicians – authors SM and RZ. If disagreements on grading of a specific sample arose, the clinicians discussed that sample and thereafter agreed upon a grade. Moreover, the optical density (OD) was measured at 548 nm (BioTek Instruments, Winooski, VT, USA) for the supernatant of duplicate samples after addition of the RGA reagents, using clear flat-bottom 96-well plates (Nunc A/S, Roskilde, Denmark) after centrifuging for 90 s at 10,000 rpm.

### In vitro analysis of subject samples

Samples were diluted 100-fold in phosphate-buffered saline (PBS-Tween, Medicago AB, Uppsala, Sweden), and 100 μL from each dilution was evenly distributed on Brucella agar plates with a cell spreader. The plates were incubated in an anaerobic chamber (COY Lab Products, Grass Lake, MI, USA. Atmosphere: 10% CO_2_, 5% H_2_ in N_2_) for 4 to 7 days in 37°C. The number of bacteria in samples from saliva, tongue scrapings and plaque, were determined by total viable counts (TVC) from plates containing 30–300 viable colonies.

### Bacteria

*In vitro* NO_2_^−^ semi-quantitation – as shown in Table 1—was conducted for various bacterial species associated with periodontitis and dental caries, as well as a selection of other bacteria. Aerobic and facultative anaerobic bacteria (except *Actinomyces* spp. and *Schaalia odontolytica*) were incubated in an aerobic box, either in 10% CO_2_ or 10% CO_2_ with N_2_ (see Table 1 for more detail), for a maximum of 7 days in 37°C. Obligate anaerobic bacteria were incubated as previously described for subject samples.

**Table 1.** Grading of NO_2_^−^ production by the RGA for a selection of bacteria.

Bacterium	Strain	Traits	Color	Grade
*Actinomyces naeslundii*	ATCC 12,104	G^+^, FA^**†**^	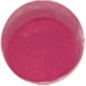	**+++**
*Schaalia odontolytica* (formerly *Actinomyces odontolyticus*)	ATCC 17,929	G^+^, FA^**†**^	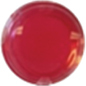	**++++**
*Actinomyces oris (*formerly *Actinomyces viscosus* serotype II)	ATCC 27,044	G^+^, FA^**†**^	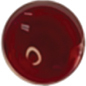	**+++++**
*Actinomyces viscosus*	ATCC 15,987	G^+^, FA^**†**^	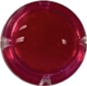	**++++**
*Aggregatibacter actinomycetemcomitans*	MW493*	G^−^, FA^**§**^	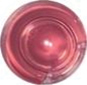	**++**
*Enterococcus faecalis*	ATCC 19,433	G^+^, FA^**‡**^	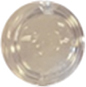	-
*Enterococcus faecium*	CCUG 542	G^+^, FA^**‡**^	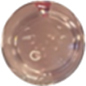	**+**
*Escherichia coli*	CCUG 21,646	G^−^, FA^**‡**^	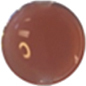	**++**
*Fusobacterium necrophorum*	CCUG 48,192	G^−^, OA^**†**^	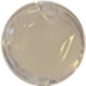	-
*Fusobacterium nucleatum*	ATCC 10,953	G^−^, OA^**†**^	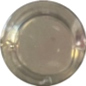	-
*Fusobacterium periodonticum*	ATCC 33,693	G^−^, OA^**†**^	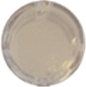	-
*Kingella denitrificans*	CCUG 14,999	G^−^, FA^**‡**^	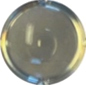	-
*Kingella kingae*	CCUG 28,875	G^−^, FA^**‡**^	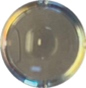	-
*Lactobacillus salivarius*	CCUG 55,845	G^+^, Ae^**‡**^	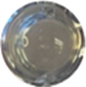	-
*Neisseria elongata*	CCUG 30,802	G^−^, Ae^**‡**^	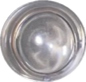	-
*Neisseria sicca/perflava*	CCUG 24,826	G^−^, Ae^**‡**^	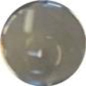	-
*Neisseria subflava*	CCUG 23,930	G^−^, MAe^**‡**^	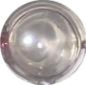	-
*Parvimonas micra*	ATCC 33,270	G^+^, OA^**†**^	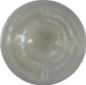	-
*Parvimonas micra*	CCUG 4635	G^+^, OA^**†**^	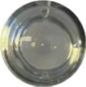	-
*Porphyromonas gingivalis*	OMGS 712	G^−^, OA^**†**^	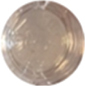	-
*Prevotella intermedia*	ATCC 25,611	G^−^, OA^**†**^	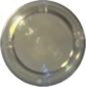	-
*Rothia dentocariosa*	CCUG 17,835	G^+^, Ae/FA^**‡**^	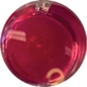	**+++**
*Rothia mucilaginosa*	CCUG 20,932	G^+^, FA^**‡**^	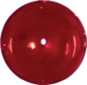	**+++**
*Staphylococcus aureus*	OMGS 3871	G^+^, FA^**‡**^	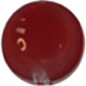	**++++**
*Staphylococcus epidermidis*	OMGS 1053	G^+^, FA^**‡**^	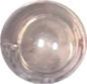	**+**
*Streptococcus anginosus*	ATCC 12,395	G^+^, FA^**‡**^	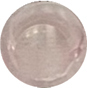	-
*Streptococcus gordonii*	OMGS 2476	G^+^, FA^**‡**^	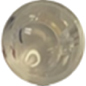	-
*Streptococcus mutans*	ATCC 25,175	G^+^, FA^**‡**^	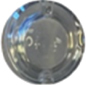	-
*Streptococcus salivarius*	CCUG 17,825	G^+^, FA^**‡**^	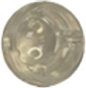	-
*Veillonella parvula*	ATCC 10,790	G^−^, OA^**†**^	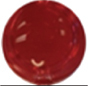	**+++++**

**CCUG**, Culture Collection, University of Gothenburg, Sweden; **ATCC**, American Type Culture Collection, USA; **OMGS**, Oral Microbiology Gothenburg Sweden, Sweden; *, clinical isolate. **G**^**+**^, Gram positive; **G**^−^, Gram negative; **FA**, Facultative anaerobic; **OA**, Obligate anaerobic; **MAe**, Microaerophilic; **Ae**, Aerobic. **†**, Anaerobic culturing with 10% CO_2_ and 5% H_2_ in N_2_; **‡**, Aerobic culturing with 10% CO_2_; **§**, Aerobic culturing with 10% CO_2_ in N_2_.

### RGA scale

The RGA Scale was based on visual grading of subject samples and control against OD (described above), as well as a standard series of known levels of Ca(NO_2_)_2_ (Sigma Aldrich, Burlington, MA, USA) in NO_3_^−^ broth. The standard was distributed two-dimensionally on a 96-well plate and ranged from 9080 μM to 0.1 μM NO_2_^−^ using 35 wells, plus seven control wells with no added Ca(NO_2_)_2_.

### Statistical analyses

*GraphPad Prism 10.0.3 (217)* was used for statistical analyses. The Shapiro–Wilk test for normality was employed on TVC, leading to the decision of utilizing non-parametric statistical tests in this study. Kruskal–Wallis test with Dunn’s multiple comparisons test, two-way Spearman correlation, as well as Friedman’s test were employed, as presented in the results section. Results were considered statistically significant when *p* < 0.05.

## Results

### RGA detection range

The lowest detected NO_2_^−^ concentration measured by the RGA was 6 μM (Figure 1), determined using the Ca(NO_2_)_2_ standard series. Figure 1 presents both the RGA grading scale (grades 0–5) and an RGA interpretation scheme for the grades: none (grade 0), low (grades 1–2), medium (grade 3) and high (grades 4–5).

The majority of the Griess reaction occurred within 2 min after sample application to the RGA solution. The stability of the visual color change was monitored for 20 min and remained stable.

### RGA reliability

**Figure 2.**
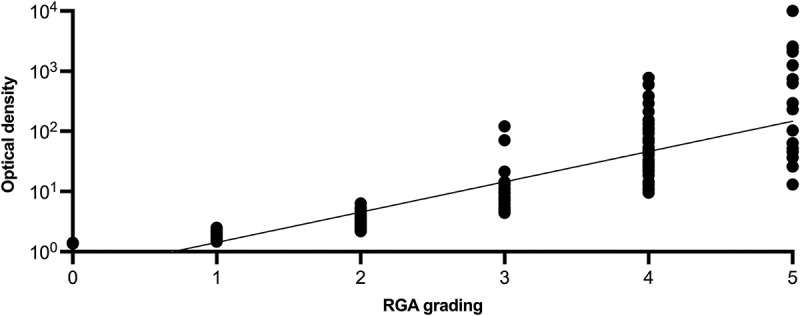
RGA grading in relation to OD. The data presented does not take sample site into account. The figure illustrates the positive correlation between OD and RGA. Note that OD is presented in a logarithmic scale.

Figure 2 reveals a strong positive correlation between OD and RGA grading. Overlap between grades occurs for low, as well as higher grades, and the degree of overlap is similar between grades. The positive relationship between OD and RGA grade was verified using Spearman’s ρ = 0.9246 with *p* < 0.0001, which was statistically significant.

### In vitro evaluation of bacterial NO_2_^−^ production

Among the 30 bacterial strains tested, maximum and minimum reaction (grade 5 and 0 respectively), were found for strains having Gram positive, Gram negative, obligate anaerobic and facultative anaerobic characteristics (Table 1). From the periodontitis associated bacteria *Porphyromonas gingivalis, Prevotella intermedia* and *Fusobacterium* spp. no NO_2_^−^ was detected (grade 0). *Aggregatibacter actinomycetemcomitans*, however, was positive for NO_2_^−^ production. Except for *Veillonella parvula*, the same was observed for the dental caries associated bacteria *Streptococcus mutans and Lactobacillus salivarius*.

All *Actinomyces* strains tested (including *S. odontolytica*, formerly known as *Actinomyces odontolyticus*) and *V. parvula* showed RGA grades 3–5.

### Ex vivo sample evaluations

**Figure 3.**
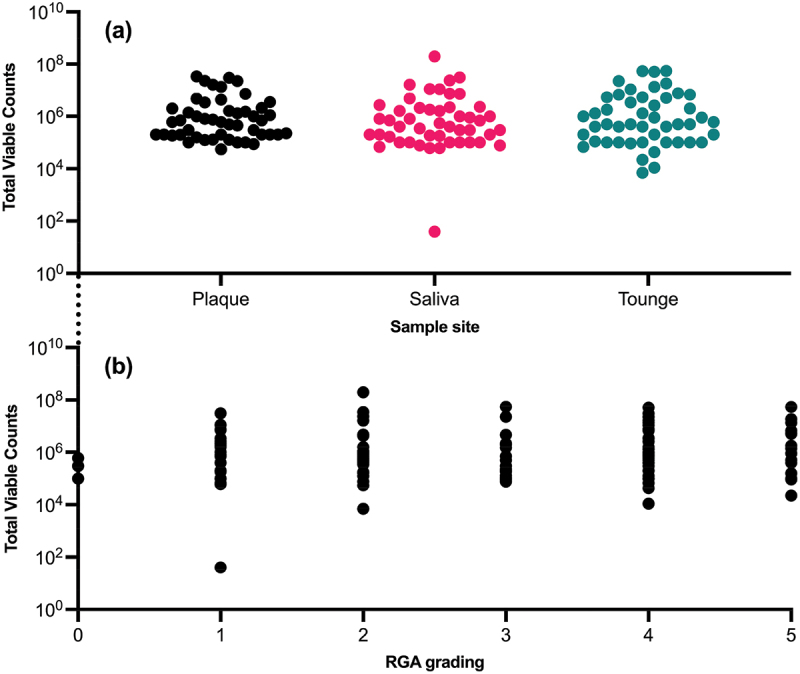
TVC from all samples taken in this study. (a) depicts samples from different sample sites and the corresponding TVC. (b) shows the RGA grading of each sample and their corresponding TVC. Each dot represents one sample. Note that TVC is depicted in log_10_.

As evident from Figure 3, all samples taken – regardless of sample site and not taking sample donor into account – showed a uniform, although wide, variation in TVC ranging from 5 log_10_ to 8 log_10_. For plaque, the median (given in log_10_) was 5.8 (IQR = 1.0), for saliva 5.7 (IQR = 1.3) and for tongue samples 5.7 (IQR = 1.7), respectively. The Kruskal–Wallis test indicated that the samples from the different sites were comparable regarding TVC (*p* = 0.8153).

Moreover, Figure 3 also depicts that the RGA grading is not a function of TVC. This is supported by a statistically non-significant relationship between the two variables with Spearman’s ρ = 0.09014 and *p* = 0.2826.

### NO_2_^−^ production capacity measured by RGA

**Figure 4.**
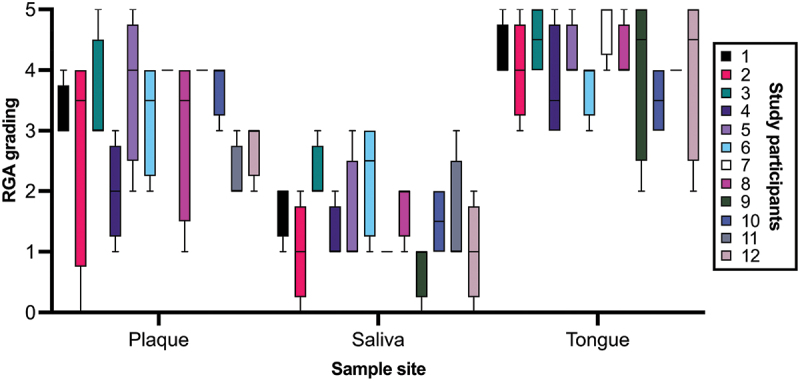
RGA grading for all study participants and all sample sites over the course of four weeks. Note that saliva samples lie in the lower region, tongue samples lie in the higher region, and plaque samples varies the most.

Through the 4 weeks of sampling, tongue samples produced the highest NO_2_^−^ concentrations with the highest long-term stability (median = 4, IQR = 0.44). Saliva samples showed the weakest NO_2_^−^ concentrations with medium long-term stability (median = 1.5, IQR = 0.75). Plaque samples showed medium-to-high NO_2_^−^ concentrations with the lowest stability over time (median = 3.25, IQR = 1). In Figure 4, the larger range in RGA grading of plaque samples – both between individual subjects as well as within the same subject over time – is depicted. The Kruskal–Wallis test reviled a statistically significant difference between sample sites with *p* < 0.0001. Furthermore, Dunn’s multiple comparisons test revealed a statistically significant difference between plaque and saliva, tongue and saliva, but not plaque and tongue (*p* = 0.0090, *p* < 0.0001, and *p* = 0.0651, respectively).

It is worth noting that subject 7 produced the same result for all plaque and saliva samples, respectively – the tongue samples varied one grade across the 4 weeks. Subject 9 presented identical RGA grading on all plaque samples, and subject 11 produced the same RGA grading for all tongue samples.

When comparing samples from different weeks within individuals using Friedman’s test, no statistical significance was found for plaque and tongue samples (*p* = 0.3449 and *p* = 0.6007 respectively). Saliva samples indicated differences between weeks with *p* = 0.0166, but Dunn’s multiple comparisons test did not yield any statistically significant difference.

### Interpretation of samples by RGA

**Figure 5.**
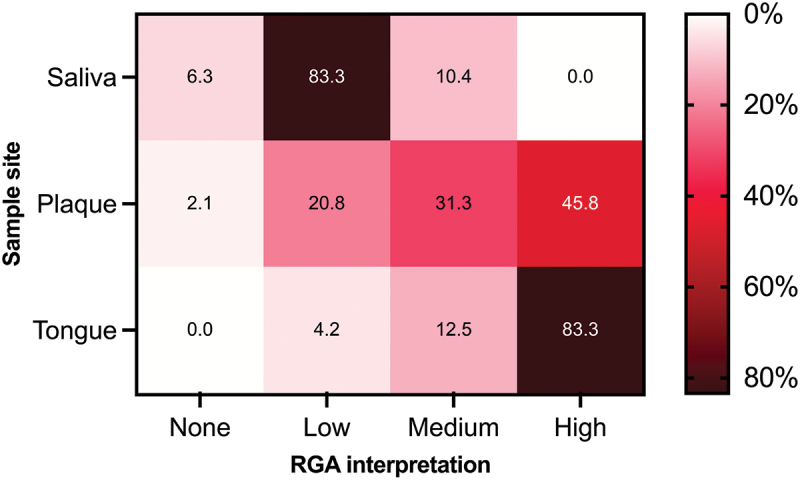
Percentage of samples shown as RGA interpretation by sample site. All values are expressed in percentage of samples within each sample site.

In Figure 5, samples are categorized using the RGA interpretation scheme (i.e. ‘none’, ‘low’, ‘medium’, and ‘high’, as illustrated in Figure 1). The heat map displays the percentage distribution of samples across each sample site.

Tongue samples showed high NO_2_^−^ production (83%), saliva samples showed low NO_2_^−^ production (83%), whereas plaque samples were highly variable with distribution of 21%, 31% and 46% for low, medium and high NO_2_^−^ production, respectively.

## Discussion

NO_2_^−^ quantitation is a difficult, expensive and time-consuming endeavor, requiring pre-treatment of samples, and hitherto, no chairside semi-quantitative method for oral NO_2_^−^ production in saliva, plaque and tongue samples – as far as we know – has been presented in the available literature. Therefore, in this pilot study, we present a reliable chairside method for semi-quantitation of oral NO_2_^−^ production from saliva, plaque and tongue samples, based on the Griess diazotization reaction. Using this method, we show that oral NO_2_^−^ concentrations are highest on the tongue dorsum, lowest in saliva and variable in dental plaque.

Johann Peter Griess presented the discovery of diazonium compounds in 1858 and further elaborated on these substances and their applications in 1864 [18,19]. Moreover, in 1879, Griess applied diazotization for the detection of NO_2_^−^ in saliva [16]. The reagents used in this reaction have since been modified many times over [13]. Also, advanced methods such as spectrophotometry, liquid- and gas chromatography, chemiluminescence, mass-spectrometry and more are often used for sensitive NO_2_^−^ quantitation. First, however, sample pre-treatments are performed in order to reduce interference and increase the specificity and sensitivity of the sample analysis [20]. Therefore, Liu and colleagues advocate the development and use of simple methods, enabling rapid and large-scale monitoring with satisfactory results [20].

The method presented in this paper – the RGA – provides the features demanded for large-scale, reproducible, stable and rapid results, utilizing a simple and cheap NO_2_^−^ semi-quantitation approach from oral samples. Primarily, the method is useful for large-scale determination of high, medium or low oral NO_2_^−^ producing abilities. This in turn enables handling and direct analysis of large quantities of data, which may prove useful in clinical applications, as well as large-scale investigations under field conditions.

Our results showed that NO_2_^−^ in supragingival dental plaque samples varied more between as well as within individuals over time than saliva and tongue samples. This may be a consequence of the ever-changing milieu of supragingival plaque: for instance, through near-time consumption of nutrients and foodstuffs or mechanical disruption of plaque through brushing, affecting the construction of the plaque. Moreover, the collection of supragingival plaque is a difficult exercise, possibly more prone to operator error as well as variation across different loci in the mouth.

Tongue samples showed the highest and stable results within and between individuals over time, with a notably lower IQR than plaque formation. The notion that NO_2_^−^ production occurs on the tongue dorsum, primarily springs from early studies in rats (germ-free and not) [21]. Later, the tongue was reported to also present the highest relative abundance of NO_2_^−^ producing bacteria and highest NO_2_^−^ production, as opposed to the rest of the oral cavity [11]. In a recent study, however, saliva was found to harbor a higher relative abundance of NO_3_^−^ reducing species, with the authors discussing whether difficulty and variability in tongue sampling might explain this new finding [22]. Still, the tongue dorsum was shown to have a higher proportion of *Streptococcus* spp., *Actinomyces* spp. and *Rothia* spp., among others. Previously, Doel et al. presented *Veillonella* spp., *Actinomyces* spp. and *Rothia* spp. as potent NO_3_^−^ reducers residing on the tongue dorsum, which is in line with our observations of these bacteria as high and medium NO_2_^−^ producers [11]. Our finding of higher NO_2_^−^ production in tongue samples may therefore be reasonably explained by either higher relative abundance of NO_2_^−^ producing species, higher NO_2_^−^ producing capacity on the tongue, or both.

In a similar manner, saliva samples remained stable over time (less so than tongue samples, but notably more than plaque samples), however with low NO_2_^−^ concentrations. This may be explained by the salivary bacterial composition emanating from the entire oral cavity. Moreover, since the crypts of the dorsum of the tongue are not easily accessible for salivary flow, the potent tongue NO_2_^−^ producers might prove less frequent in saliva, perhaps resulting in lower NO_2_^−^ production.

The RGA proved especially useful regarding NO_2_^−^ concentrations when applying the interpretation scheme presented in Figure 1 (data presented in Figure 5). This further underlines the differences in NO_2_^−^ concentrations throughout the oral cavity, and also indicates that one may expect NO_2_^−^ to be present in the oral cavity of every subject (note that only 4 of 144 samples produced RGA grading of 0).

An important note is also that the samples collected contained similar TVC (see Figure 3 (a) and median values for the sample sites), indicating that the NO_2_^−^ produced was not a function of the bacterial abundance, but rather the NO_2_^−^ present in different samples. This further shows that some NO_2_^−^ producing species are more potent NO_2_^−^ producers than others and potentially can compensate for lower abundance (as previously discussed).

Several publications have reported on the NO_3_^−^ reducing abilities of several oral bacteria – notably, *Rothia* spp., *Neisseria* spp., *Actinomyces* spp., *Veillonella* spp. and *S. odontolytica* [11,12,23]. In identifying these bacteria as NO_3_^−^ reducing species, methods based on the Griess reaction have often been utilized. It is then important to point out that the Griess reaction involves the coupling of reagents to NO_2_^−^. Consequently, it is not a method suitable for determining NO_3_^−^ reduction where other potential metabolites may be the end product (notably NO, N_2_O, NH_4_^+^and N_2_) [4,13,24,25]. We deem this to be an important point for two main reasons. First, it is unclear whether it is the orally produced NO_2_^−^, NO or both that may be associated with potential health benefits. Second, bacterial species may possess and express genes for nitrate reductases (NR), nitrite reductases (NiR) and nitric-oxide reductase (NOR) simultaneously or separately [26–28]. This naturally becomes even more complex in an *in vivo* biofilm environment. When measuring the amount of NO_3_^−^ reduced, uncertainty remains regarding reduction to metabolites beyond NO_2_^−^ in the reduction chain. Therefore, a focus on oral NO_2_^−^ production capacity provides higher specificity and limits misconceptions.

Importantly then, is that there are multiple potential mechanisms through which oral NO_2_^−^ can materialize. Some examples are: through oxidation of NO in the L-arginine-pathway, through reduction of NO_3_^−^ to NO_2_^−^ by Xanthine oxidase, and through the bacterial reduction of NO_3_^−^ to NO_2_^−^ by NR enzymes [10,11,29–31]. The RGA semi-quantitates NO_2_^−^ content in oral samples and therefore is an assay for the total oral NO_2_^−^ production—i.e. NO_2_^−^ generation regardless of mode of production, including the ability to reduce NO_3_^−^ to NO_2_^−^, while simultaneously excluding further metabolites of NO_2_^−^.

The RGA semi-quantitates the bacterial NO_2_^−^ producing capacity from NO_3_^−^ in conjunction with circulating NO_2_^−^ from saliva from oral samples. *In vitro*, however, the RGA is a true semi-quantitative measure of bacterial NO_2_^−^ production.

We found that *A. oris, A. viscosus, S. odontolytica*, *V. parvula, and Staphylococcus aureus* were powerful NO_2_^−^ producers. This is largely in accordance with the findings of Sato-Suzuki and coworkers., in which several of these species constituted a large portion of NO_2_^−^ producers on the tongue [12]. Regarding *Neisseria* spp., which has been cited in the literature as important and potent NO_2_^−^ producers, we could not identify any NO_2_^−^ producing activity, which contrasts with the findings of one study [12], but is in agreement with others [32,33]. Since several species of *Neisseria*– in addition to NR genes or absence of them – also possess NiR genes, NO_2_^−^ is probably not detected due to its rapid further reduction or since no NO_2_^−^ is being produced [26,28]. It is also worth noting that many *Neisseria* spp. seemingly have a stronger correlation to NO_2_^−^ reduction than NO_3_^−^ reduction [32–34]. This was presented in a previous investigation of some *Neisseria* spp. showing NiR genes in all the tested strains [28]. Therefore, it is likely that the contribution of *Neisseria* spp. to the overall oral NO_2_^−^ concentration, is limited. This supports our finding of no NO_2_^−^ production by *Neisseria elongata, Neisseria sicca/perflava* and *Neisseria subflava* in this investigation. These findings furthermore underline the importance of phenotypic investigations, since the assumption of NO_2_^−^ production by *Neisseria* spp. (among others) primarily is based on findings from molecular techniques for genotyping in which NR-genes have been identified.

*A. actinomycetemcomitans* produced low concentrations of NO_2_^−^ when evaluated with the RGA. NR-genes have been located in strains of *A. actinomycetemcomitans*, supporting the notion that the bacterium may be able to produce NO_2_^−^ [35].

Both *Kingella kingae* and *Kingella denitrificans* showed no NO_2_^−^ production. This also is in accordance with previous publications, where *K. kingae* did not reduce NO_3_^−^- nor NO_2_^−^, and *K. denitrificans* reduced both [32,36]. For *K. denitrificans*, zinc dust was added to the test solution as a reducing agent of NO_3_^−^, to evaluate if NO_3_^−^ had been reduced. The color remained unchanged, suggesting that there was neither NO_3_^−^ nor NO_2_^−^ in the sample. Thus, it is probable that *K. denitrificans* does indeed reduce NO_3_^−^ to NO_2_^−^, but that the reduction continues with the reduction of NO_2_^−^ to other metabolites, wherefore the NO_2_^−^ produced is not detected by the RGA.

*Rothia dentocariosa* and *Rothia mucilaginosa*, which are also associated with NO_2_^−^ production and health, according to the literature, were found to be NO_2_^−^ producing with a medium production capacity [4,11,37].

## Conclusion

In conclusion, the presented method – RGA – for oral *ex vivo* NO_2_^−^ semi-quantitation is a quick, simple and reliable tool for chairside use in clinical or research application. It allows for large-scale sampling and analysis in a matter of minutes.

Healthy individuals differ in their orally produced NO_2_^−^ concentrations. The dorsum of the tongue seems to provide potent NO_2_^−^ production capacity across individuals, as well as within individuals over time. Likewise, saliva provides low but consistent NO_2_^−^ production capacity between and within individuals over time.

The oral bacterial species *Actinomyces* spp., *Rothia* spp., *V. parvula* and *A. actinomycetemcomitans* showed NO_2_^−^ production with RGA method.
